# Discontinuation due to immune‐related adverse events is a possible predictive factor for immune checkpoint inhibitors in patients with non‐small cell lung cancer

**DOI:** 10.1111/1759-7714.13149

**Published:** 2019-07-22

**Authors:** Kazutoshi Komiya, Tomomi Nakamura, Tomonori Abe, Shinsuke Ogusu, Chiho Nakashima, Koichiro Takahashi, Shinya Kimura, Naoko Sueoka‐Aragane

**Affiliations:** ^1^ Division of Hematology, Respiratory Medicine and Oncology, Faculty of Medicine Saga University Saga Japan

**Keywords:** Immune checkpoint inhibitor, immune‐related adverse event, interstitial lung disease, non‐small cell lung cancer, predictive factor

## Abstract

**Background:**

Immune‐related adverse events (irAEs) should be anticipated with treatment by immune checkpoint inhibitors (ICIs). Although the relationship between irAEs and efficacy of ICI has been reported, it has not yet been clarified whether the benefit from ICI outweighs the low frequency of proceeding to subsequent therapies after discontinuation due to irAEs.

**Methods:**

The study comprised 61 patients with non‐small cell lung cancer who underwent treatment with ICIs (nivolumab or pembrolizumab monotherapy) at the Saga University Medical School Hospital from December 2015 to January 2018. Therapeutic effect and progression‐free survival (PFS) were compared between the irAEs discontinuation group (AEg) and the group with discontinuation due to all causes other than irAEs (Non‐AEg).

**Results:**

A total of 30% patients(18/61) had therapy discontinued due to irAEs: 22.5% (9/40) with nivolumab and 42.9% (9/21) with pembrolizumab. The response rate was 50.0% in the AEg and 8.1% in the on‐AEg (*P* = 0.001). The median PFS was significantly longer in the AEg (9.3 months; 95% CI 2.1–12.1) than in the non‐AEg (1.9 months; 95% CI 0.9–3.6): HR 0.45 (95%CI 0.20–0.89; log‐rank test *P* = 0.026). The prevalence of drug‐induced interstitial lung disease (ILD) was 6.1% (3/49) in cases without interstitial pneumonia (IP) as the underlying disease, whereas it was 50% (6/12) in cases with IP (*P* = 0.001).

**Conclusion:**

Discontinuation of treatment with ICIs due to irAEs predict a good response to ICIs and favorable outcome since their anti‐cancer effects continue even after discontinuation. However, the presence of IP as the underlying disease increases the risk of drug‐related ILD onset.

## Introduction

In recent years, immune checkpoint inhibitors (ICIs) have achieved prominence in the treatment of advanced or recurrent non‐small cell lung cancer (NSCLC). Several phase III trials showed prolonged overall survival with ICIs in patients with NSCLC who were previously untreated or treated with alternative therapies.^1^, ^2^, ^3^, ^4^ Although it was concluded that the safety profile was good in all these trials, there were also serious immune‐related adverse events (irAEs), which is one of the primary reasons for discontinuing ICIs. In the international phase III trials of nivolumab, discontinuation due to treatment‐related AEs occurred in 3% of patients in the CheckMate 017 study and in 5% of patients in the CheckMate 057 study; among the discontinued cases, pneumonitis was the most common treatment‐related AE.^1^, ^2^ In KEYNOTE‐024 and KEYNOTE‐021—the international phase III trials of pembrolizumab—4.0–7.1% of patients had therapy discontinued due to treatment‐related AEs.^3^, ^4^ In addition, frequency of proceeding to subsequent therapies was lower than with chemotherapy.^5^

An association between occurrence of irAEs and treatment outcome with immunotherapy has also been previously reported. In a retrospective study, overall response rate (ORR) and median progression‐free survival (PFS) were better in patients with irAEs of grade 3 or higher than in patients with grade less than 3: 25% vs. 6% and 30 weeks vs. 10 weeks, respectively.^6^ Similar results were seen in a retrospective study from a Japanese institution, in which the efficacy and prognosis were better in the irAE group: ORR was 57% in the irAE group and 12% in the non‐irAE group, PFS was 12.0 months in the irAE group and 3.6 months in the non‐irAE group.^7^ Other retrospective studies of NSCLC patients who received nivolumab have also reported an association between irAEs and treatment outcome.^8^, ^9^ As described above, occurrence of irAEs was deemed to be useful as a predictive and prognostic factor for judging success of immunotherapy. An important clinical question is whether the benefit from ICI overcomes the low frequency of proceeding to subsequent therapies after discontinuation due to irAEs. Therefore, we evaluated detailed clinical courses after discontinuation of ICI due to irAEs, and compared efficacy and prognosis between the group of cases with treatment discontinued due to irAEs and those discontinued for other reasons. In addition, we were able to examine the risk of drug‐related interstitial lung disease (ILD) in patients with a history of ILD, which is cause for exclusion from many clinical trials.

## Methods

### Patients

A total of 61 patients were studied who had NSCLC and received either nivolumab or pembrolizumab at Saga University Medical School Hospital between December 2015 and January 2018. The AE group (AEg) was defined as those whose treatment administration was discontinued due to irAEs, and the non‐AE group (Non‐AEg) was defined as those whose administration was discontinued for all other reasons. We retrospectively examined the efficacy, prognosis, and safety of anti‐programmed death‐1 (PD‐1) antibody therapy. The cutoff for data collection was 30 June 2018. The study protocol was approved by the Saga University Clinical Research Ethics Committee. All patients gave informed consent for the research use of tissue and cytology specimens.

### Outcome assessment

Expression of programmed death‐ligand 1 (PD‐L1) was examined by immunohistochemical staining by using PD‐L1 IHC 22C3 pharmDx. Tumor response was evaluated according to Response Evaluation Criteria In Solid Tumors (RECIST) version 1.1. The severity of irAEs was graded on the basis of the Common Terminology Criteria for Adverse Events (CTCAE) version 4.0.

### Statistical analysis

For testing the difference of clinical background between the AEg and the Non‐AEg, a Chi‐square test or Mann‐Whitney U test was used. The survival rate was calculated according to the Kaplan‐Meier method and the log‐rank test was used for assessing differences. Cox proportional hazards regression analysis, with adjustment for the potentially confounding variables age, smoking index, gender, histology, and the Eastern Cooperative Oncology Group performance status (ECOG PS), was used to calculate the hazard rate (HR) and 95% confidence intervals (CI). *P*‐values less than 0.05 were regarded as statistically significant. Statistical analyses were conducted using SPSS statistics 19 software (SPSS Japan Inc. Tokyo) and JMP Pro 13 software (SAS Institute Inc., USA).

## Results

### Patient characteristics

Among the 61 patients undergoing anti‐PD‐1 antibody therapy, 40 were treated with nivolumab and 21 with pembrolizumab (Table 1). Median age at start of treatment was 70 years in the nivolumab group and 69 years in the pembrolizumab group. Most patients were men (89%), were smokers with smoking index greater than 400 (87%), had nonsquamous cell carcinoma (64%), and had ECOG PS 0–1 (82%). Among 52 patients with whom we were able to perform expression analysis of PD‐L1, 23 (44%) were found to express at level 50% or more, 17 (33%) were at levels 1–49%, and 12 (23%) were at levels less than 1%. There were six patients (15%) in the nivolumab group and 6 (29%) in the pembrolizumab group with preexisting ILD.

**Table 1:** Clinicopathological characteristics of the patients

	Nivolumab (*n* = 40)	Pembrolizumab (*n* = 21)
Median age, years (range)	70 (44–81)	69 (48–82)
Sex		
Male / Female	34/6	20/1
Brinkman index		
≧400/<400	35/5	18/3
Histology		
Squamous/Nonsquamous	16/24	6/15
Stage		
up to III/IV/recurrence	11/20/9	7/10/4
ECOG PS		
0 / 1 / 2+	14/18/8	7/11/3
PD‐L1 expression (%)		
0 / 1–49/50 + / n.d.	12/14/5/9	0/3/18/0
Treatment line		
1/2+	0/40	14/7
Preexisting IIP or radiation pneumonitis		
Y/N	6/34	6/15

ECOG, Eastern Cooperative Oncology Group; IIP, idiopathic interstitial pneumonia; n.d., not determined; PD‐L1, programmed death‐ligand 1; PS, performance status;

### Treatment efficacy and discontinuation rate

The therapeutic response rate was 22.5% in the 40 patients who received nivolumab and 42.9% in the 14 patients who received pembrolizumab in the first line therapy, and the disease control rates were 60.0% and 71.5%, respectively (Table 2). Discontinuation due to treatment‐related irAEs occurred in 22.5% of the nivolumab group versus 42.9% of the pembrolizumab group, but the difference was not statistically significant. In the group treated with nivolumab, there were two occurrences of ILD, two of colitis/diarrhea, and one each of ILD/myalgia, fever/malaise/anorexia, diverticulitis, tuberculosis, and skin disorder. In the group treated with pembrolizumab, there were four occurrences of ILD and one each of hemoptysis/hematemesis, ILD/atelectasis, isolated ACTH deficiency, rash/fever/diarrhea/hyperthyroidism, and hypothyroidism. The prevalence of irAEs was 32.4% in the Non‐AEg. The majority of ICI discontinuation in the Non‐AEg was due to disease progression (83.8%); the other reason was general deterioration of physical condition.

**Table 2:** Response and discontinuation rate by cause

		Pembrolizumab (*n* = 21)		
	Nivolumab (*n* = 40)	1st line (*n* = 14)	Second and subsequent line (*n* = 7)	*P*‐value
Best overall response				
Complete response (CR)	0 (0%)	0 (0%)	0 (0%)	
Partial response (PR)	9 (22.5%)	6 (42.9%)	1 (14.3%)	
Stable disease (SD)	15 (37.5%)	4 (28.6%)	2 (28.6%)	
Progressive disease (PD)	10 (25%)	3 (21.4%)	4 (57.1%)	
Not evaluable (NE)	6 (15%)	1 (7.1%)	0 (0%)	
Overall response rate (ORR)	9 (22.5%)	6 (42.9%)	1 (14.3%)	
Disease control rate (DCR)	24 (60%)	10 (71.5%)	3 (42.9%)	
Discontinuation due to irAEs	9 (22.5%)	9 (42.9%)		0.140*
Discontinuation due to non‐irAEs	27 (67.5%)	10 (47.6%)		0.171*

* Fisher's exact test.

irAE, immune‐related adverse event.

### Clinical course of irAEs discontinuation group and prognostic analysis

**Figure 1:**
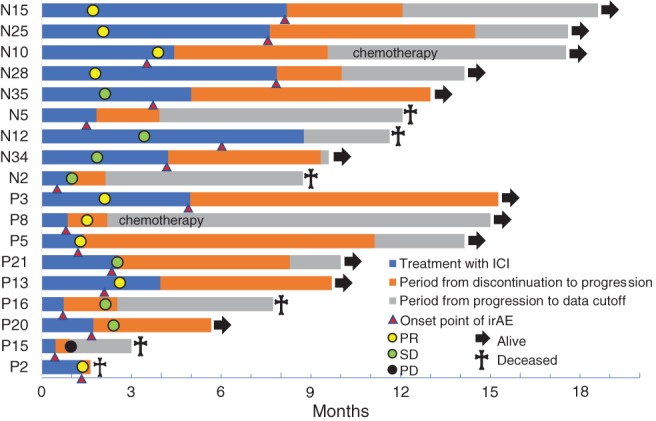
Clinical course of patients who had therapy discontinued due to irAEs. N represents nivolumab administration; P represents pembrolizumab administration. After discontinuation of ICIs, only two patients were given subsequent treatment.

Between the AEg and on‐AEg groups, there was no significant difference in age, sex, smoking index, histology, ECOG PS, or treatment duration (Table 3). PD‐L1 expression was slightly higher in the AEg, but the difference was not statistically significant. The response rate in the AEg (50.0%) was higher than that in the Non‐AEg (8.1%; *P* = 0.001). Similarly, the disease control rate was significantly higher in the AEg (94.4% vs. 37.8%; *P* < 0.001). The clinical course from the start of administration of anti‐PD‐1 antibody in each case of the AEg is shown in Figure 1. The irAEs and their severity leading to treatment discontinuation are shown in Table S1. Four patients had sustained therapeutic benefit for more than six months after discontinuation due to irAEs (patients N25, N35, P3, and P5). In addition, even if disease progression occurred after discontinuation due to irAEs, follow‐up was possible for more than four months without treatment in five cases (patients N15, N28, N5, N2, and P16). The median period from discontinuation to progression was 117 days (range, 1 to 309).

**Table 3:** Comparison of clinical background between the AEg and the on‐AEg

	AEg (*n* = 18)	Non‐AEg (*n* = 37)	*P*‐value
Median age (range)	69 (45–79)	71 (48–82)	0.524
Sex			
Male	17	31	0.406
Female	1	6	
Brinkman index, median (range)	1010 (62–2400)	1000 (0–2400)	0.836
Histology			
Squamous	5	15	0.391
Nonsquamous	13	22	
ECOG PS			
0 or 1	16	29	0.470
2	2	8	
PD‐L1(%)†, median (range)	65 (0–100)	20 (0–100)	0.175
Best overall response			
CR/PR (ORR %)	9 (50.0%)	3 (8.1%)	0.001
SD/PD/NE	9	34	
CR/PR/SD (DCR %)	17 (94.4%)	14 (37.8%)	<0.001
PD/NE	1	23	
Treatment duration (days), median (range)	98 (14–263)	46 (0–384)	0.798*

* Log‐rank test.

† Evaluable cases only.

CR, complete response; DCR, disease control rate; ECOG, Eastern Cooperative Oncology Group; NE, not evaluable; ORR, overall response rate; PD‐L1, programmed death‐ligand 1; PD, progressive disease; PR, partial response; PS, performance status; SD, stable disease; Sq.

**Figure 2:**
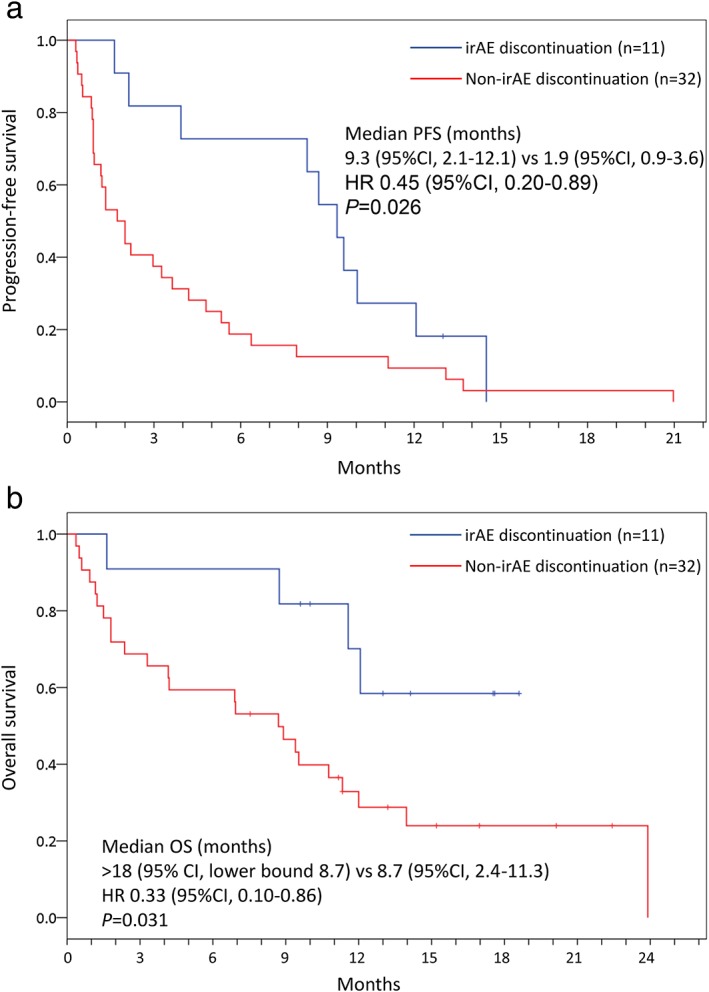
Kaplan‐Meier curves of progression‐free survival (**a**) and overall survival (**b**) in patients in whom ICI was administered as a treatment after the second line therapy.

PFS and overall survival (OS) were analyzed in 43 patients (11 patients in the AEg and 32 in the Non‐AEg) after exclusion of 12 patients who received anti‐PD‐1 antibody in the first line therapy. The median PFS was 9.3 months (95% confidence interval [CI], 2.1 to 12.1) in the AEg and 1.9 months (95% CI, 0.9 to 3.6) in the Non‐AEg: HR, 0.45; 95% CI, 0.20 to 0.89; *P* = 0.026 (Fig 2(a)). The median OS was not reached in the AEg (lower 95% confidence bound 8.7 months) and was significantly longer than that in the Non‐AEg (8.7 months; 95% CI, 2.4 to 11.3): HR, 0.33; 95% CI, 0.10 to 0.86; *P* = 0.031 (Fig 2(b)). With multivariable analysis (Table 4), discontinuation due to irAEs was revealed to be a potential prognostic factor for PFS (HR, 0.41; 95% CI, 0.18 to 0.85; *P* = 0.016) despite adjustment for the other factors.

**Table 4:** Multivariable Cox regression analysis of the hazard of disease progression

Characteristics	Hazard ratio (95% CI)	*P*‐value
Age (≧75 years vs. <75 years)	0.89 (0.42–1.79)	0.758
Brinkman index (≧400 vs. <400)	0.96 (0.32–3.27)	0.940
Sex (male vs. female)	1.16 (0.32–4.67)	0.827
Histology (squamous vs. nonsquamous)	0.65 (0.31–1.30)	0.225
ECOG PS (0/1 vs. 2/3)	0.77 (0.28–2.38)	0.631
Reason for ICI discontinuation (irAEs vs. non‐irAEs)	0.41 (0.18–0.85)	0.016

CI, confidence intervals; ECOG, Eastern Cooperative Oncology Group; ICI, immune checkpoint inhibitor; irAEs, immune‐related adverse events; PS, performance status.

The period from discontinuation to the time either the next therapy was begun or a decision was made to begin best supportive care was analyzed in those patients. The median period was 5.2 months (95% CI, 4.4 to 5.9) in the AEg and 0.5 months (95% CI, 0.44 to 0.56) in the Non‐AEg: HR, 0.09; 95% CI, 0.02 to 0.27; *P* < 0.001 (Fig. S1). With multivariable analysis (Table S2), discontinuation due to irAE was revealed to be a potential predictive factor for the period from treatment discontinuation to the next therapy or decision for best supportive care.

### History of interstitial lung disease is associated with drug‐related ILD

**Figure 3:**
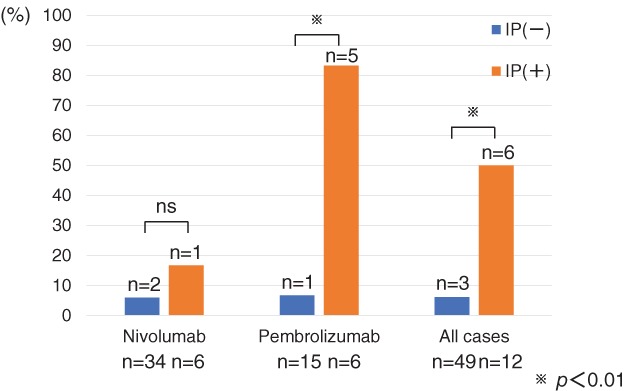
Prevalence of drug‐induced interstitial lung disease (ILD). Prevalence of drug‐induced ILD is significantly higher in patients with preexisting ILD.

There were five cases of idiopathic interstitial pneumonia and seven cases of radiation pneumonitis (12 cases in total) among the 61 patients with a history of ILD. None of the patients was undergoing systemic steroid therapy for preexisting ILD before the start of anti‐PD‐1 antibody therapy. In the nivolumab group, the prevalence of drug‐related ILD in patients without preexisting ILD was 6%, whereas in the patients with preexisting ILD, the prevalence was 17% (Fig 3), but the difference was not statistically significant. In the pembrolizumab group, the prevalence of drug‐related ILD was significantly higher in patients with preexisting ILD than in patients without preexisting ILD: 83% vs. 7%, respectively (*P* = 0.002). Even among all patients, the prevalence of drug‐related ILD was significantly higher in those with preexisting ILD (50%) than in those without (6%; *P* = 0.001). Table 5 shows the clinical features of nine patients who developed drug‐related ILD. Seven of them had events of grade 2 or less, and drug‐related ILD rapidly improved with drug withdrawal or oral corticosteroids. Patient P15, with a history of interstitial pneumonia of usual interstitial pneumonia (UIP) pattern, died of respiratory failure without responding to treatment, despite steroid pulse therapy. The response rate of the nine patients who developed ILD was 56%. There were no patients who received ICI rechallenge after the onset of drug‐related ILD.

**Table 5:** Clinical features of patients who developed immune‐related interstitial lung disease (ILD)

Pt	Line	Period until onset (days)	Grade	Response	Preexisting interstitial lung disease	Radiological pattern or finding in chest CT	Treatment (treatment period with corticosteroid, days)	Outcome
N5	2	118	2	SD	None	Focal GGO	Oral corticosteroid (139)	Improved
N15	4	259	1	PR	IIP	OP	Oral corticosteroid (88)	Improved
N28	2	247	3	PR	None	OP, fibrosis	Steroid pulse, NPPV (213)	No change
P3	1	166	1	PR	Radiation pneumonitis	Focal GGO	Drug discontinuation	Improved
P5	1	38	1	PR	IIP	Focal GGO	Oral corticosteroid (82)	Improved
P8	1	46	2	PR	IIP	OP	Oral corticosteroid (75)	Improved
P11	2	144	2	PD	Radiation pneumonitis	OP	Oral corticosteroid (86)	Improved
P15	1	14	5	PD	IIP	Bilateral diffuse GGO	Steroid pulse (76)	Death
P16	1	64	2	SD	None	OP	Oral corticosteroid (95)	Improved

CT, computed tomography; GGO, ground‐glass opacity; IIP, idiopathic interstitial pneumonia; N, nivolumab‐treated; NPPV, noninvasive positive pressure ventilation; OP, organizing pneumonia pattern; P, pembrolizumab‐treated; PD, progressive disease; PR, partial response‐; SD, stable disease.

## Discussion

We conclude that the patients who developed irAEs leading to discontinuation (AEg) had significantly better outcomes, such as ORR, PFS, and OS, than the patients with discontinuation due to other causes (Non‐AEg), and discontinuation due to irAEs was an independent prognostic factor in that it was associated with prognosis despite adjustment for other factors. Half of the patients who were discontinued due to irAEs showed prolonged progression‐free status or slow progression without further treatment. No patients in the AEg received retreatment by ICI before the cutoff for data collection. Considering retreatment by ICI, it has been reported that benefit may occur in patients who have ICI discontinued due to irAEs before tumor response has been achieved.^10^

ILD was most frequently observed among patients with irAEs. With other studies of irAEs, an association between drug‐induced ILD and treatment outcome has been reported.^11^, ^12^, ^13^ In our study, five out of nine patients (56%) who developed drug‐induced ILD had PR, and a high response rate was confirmed. However, association of the computed tomography findings of ILD with efficacy and prognosis has not been clarified. Although ICIs have been applied to treat various malignant tumors, frequency of ILD was higher in patients with lung cancer than in patients with other tumors. A review of clinical trials of patients who received ICI monotherapy reported that pneumonitis occurs more frequently in patients with lung cancer than in patients with malignant melanoma (HR2.3, 95% CI, 1.4 to 3.8), so it is speculated that irAEs are organ specific.^14^ These data suggest that irAEs affecting the patient's general condition and leading to discontinuation also lead to a strong immune response, which is considered to be related to the therapeutic effect.

Patients with a history of ILD have been excluded from many clinical trials because of a greater risk of drug‐induced ILD. However, patients with preexisting ILD are often encountered in practice, because an appropriate population for treatment with ICIs—such as smokers—contains many patients with preexisting ILD. Therefore, we examined the risk of drug‐induced ILD in patients with preexisting ILD. Our results indicate that the prevalence of drug‐induced ILD is significantly higher among patients with preexisting ILD than among those without ILD, which is similar to the results of Japanese phase II trials.^15^ According to Kanai *et al*., the prevalence of drug‐induced ILD with nivolumab is high (31%) in patients with preexisting ILD, but over 50% of patients improve during the course of therapy.^13^ Also, in a study of nivolumab in patients with radiation pneumonitis, drug‐induced ILD occurred in 26.5% of patients.^16^ On the other hand, it has been reported that there is no onset of drug‐induced ILD in patients with mild IIP.^11^ In our study, drug‐induced ILD was seen, even in patients with relatively mild IIP, so care should be taken in the administration of ICIs to such patients. Even though ILD frequently occurred with ICIs among patients with preexisting ILD in our study, all except one of those patients recovered after treatment with corticosteroid (the exception was a patient who died of ILD so that efficacy of ICI could not be observed). Evaluating the image pattern of ILD is therefore important for estimating the therapeutic reactivity to corticosteroid and prognosis.^17^ Although ILD exhibiting the organizing pneumonia pattern generally responds well to corticosteroid and has a good prognosis, ILD with the diffuse alveolar damage pattern is often accompanied by a poor prognosis.^15^, ^18^ Immunological molecular mechanisms are therefore expected to differ among seemingly similar immune‐related adverse events.

In this study, the prevalence of drug‐induced ILD in first line therapy was higher than that after second line therapy (36% vs. 9%). In a systematic review and meta‐analysis of trials on the incidence of ILD with anti‐PD‐1 antibody and anti‐PD‐L1 antibody, it was reported that treatment‐naïve patients had a significantly higher prevalence of ILD than previously treated patients (4.3% vs. 2.8%, *P* = 0.03).^19^ Thus, using ICI in first line therapy for patients with preexisting ILD may result in a high rate of drug‐induced ILD occurrence, so caution should be exercised.

Currently, PD‐L1 expression is the only predictive marker available in the clinical setting. Regardless of PD‐L1 expression, CheckMate 227, an international phase III trial of nivolumab and ipilimumab, showed benefits of immunotherapy in patients with NSCLC who had a high tumor mutation burden.^20^ Therefore, it was suggested that we could not select patients effectively by using only PD‐L1 expression. Our study showed that irAEs could be correlated with ICI efficacy and thus could predict outcome. Because ours was a single center retrospective study, the number of patients was not sufficient and discontinuation due to treatment‐related AEs and administration of ICIs to patients with a history of ILD were determined case‐by‐case by a number of different doctors. It is therefore necessary to confirm the association between irAEs and the effect of ICIs in a standardized setting. It has recently been reported that soluble PD‐1 and PD‐L1, and neutrophil‐to‐lymphocyte ratio, are useful as predictive or prognostic markers.^21^, ^22^, ^23^, ^24^ The combination of these clinical features, including irAEs with PD‐L1 expression, might lead to more precise prediction of ICI efficacy.

In summary, irAEs are one of the primary causes of discontinuation of ICIs. In our study, the efficacy of ICIs and the prognosis were significantly better in patients with discontinuation due to irAEs than in patients with discontinuation due to other reasons, which suggests that discontinuation due to irAEs might be useful as a prognostic factor. In addition, history of ILD is associated with drug‐induced ILD, so adequate risk assessment is indispensable, especially when considering administration of ICIs in first line therapy.

## Disclosure

The authors report no conflict of interest in this work.

## Supporting information


**Supplementary table 1**. Severity of immune‐related adverse events (irAEs) leading to treatment discontinuation
**Supplementary table 2**. Multivariable analysis of period from discontinuation to either next therapy or decision for best supportive care

**Supplementary figure 1**. Kaplan‐Meier curves of period from discontinuation to the next therapy or decision for best supportive care in whom ICI was administered as a treatment after the second line therapy.
