# Supplementary material for: Discontinuation due to immune‐related adverse events is a possible predictive factor for immune checkpoint inhibitors in patients with non‐small cell lung cancer
Source: Thorac Cancer. 2019 Jul 22;10(9):1798–804. doi: 10.1111/1759-7714.13149 (PMC6718019; doi:10.1111/1759-7714.13149)
Supplement: Supplementary file 1 — Supplementary table 1. Severity of immune‐related adverse events (irAEs) leading to treatment discontinuation Supplementary table 2. Multivariable analysis of period from discontinuation to either next therapy or decision for best supportive care [file TCA-10-1798-s001.docx]

Supplementary table 1

Severity of immune-related adverse events (irAEs) leading to treatment discontinuation

| Pt | irAE | CTCAE Grade |
| --- | --- | --- |
| N15 | ILD | 1 |
| N25 | tuberculosis | 2 |
| N10 | colitis | 3 |
| N28 | ILD | 3 |
| N35 | skin disorder | 2 |
| N5 | ILD, myalgia | 2 |
| N12 | diverticulitis | 2 |
| N34 | diarrhea | 2 |
| N2 | fever, malaise, anorexia | 2 |
| P3 | ILD, atelectasis | 1 |
| P8 | ILD | 2 |
| P5 | ILD | 1 |
| P21 | isolated ACTH deficiency | 2 |
| P13 | rash, fever, diarrhea, hyperthyroidism | 2 |
| P16 | ILD | 2 |
| P20 | hypothyroidism | 2 |
| P15 | ILD | 5 |
| P2 | hemoptysis, hematemesis | 5 |

Abbreviations: N, nivolumab treated; P, pembrolizumab treated; CTCAE, the Common Terminology Criteria for Adverse Events; ILD, interstitial lung disease

Supplementary table 2

Multivariable analysis of period from discontinuation to either next therapy or decision for best supportive care

| Characteristics | Hazard ratio (95% CI) | *P*-value |
| --- | --- | --- |
| Age (≧ 75 years vs ＜75 years) | 1.23 (0.60-2.41) | 0.556 |
| Brinkman index (≧400 vs ＜400) | 0.82 (0.31-2.45) | 0.715 |
| Sex (male vs female) | 1.49 (0.49-5.24) | 0.494 |
| Histology (sq vs non-sq) | 1.82 (0.91-3.68) | 0.091 |
| ECOG PS (0/1 vs 2/3) | 0.42 (0.17-1.10) | 0.074 |
| Reason for ICI discontinuation (irAEs vs non- irAEs) | 0.08 (0.02-0.24) | <0.001 |

Abbreviations: sq, squamous cell carcinoma; ECOG, Eastern Cooperative Oncology Group; PS, performance status; ICI, immune checkpoint inhibitor; irAEs, immune-related adverse events; CI, confidence intervals.
