# Supplementary material for: Discontinuation due to immune‐related adverse events is a possible predictive factor for immune checkpoint inhibitors in patients with non‐small cell lung cancer
Source: Thorac Cancer. 2019 Jul 22;10(9):1798–804. doi: 10.1111/1759-7714.13149 (PMC6718019; doi:10.1111/1759-7714.13149)
Supplement: Supplementary file 2 — Supplementary figure 1. Kaplan‐Meier curves of period from discontinuation to the next therapy or decision for best supportive care in whom ICI was administered as a treatment after the second line therapy. [file TCA-10-1798-s002.pptx]

## Slide 1
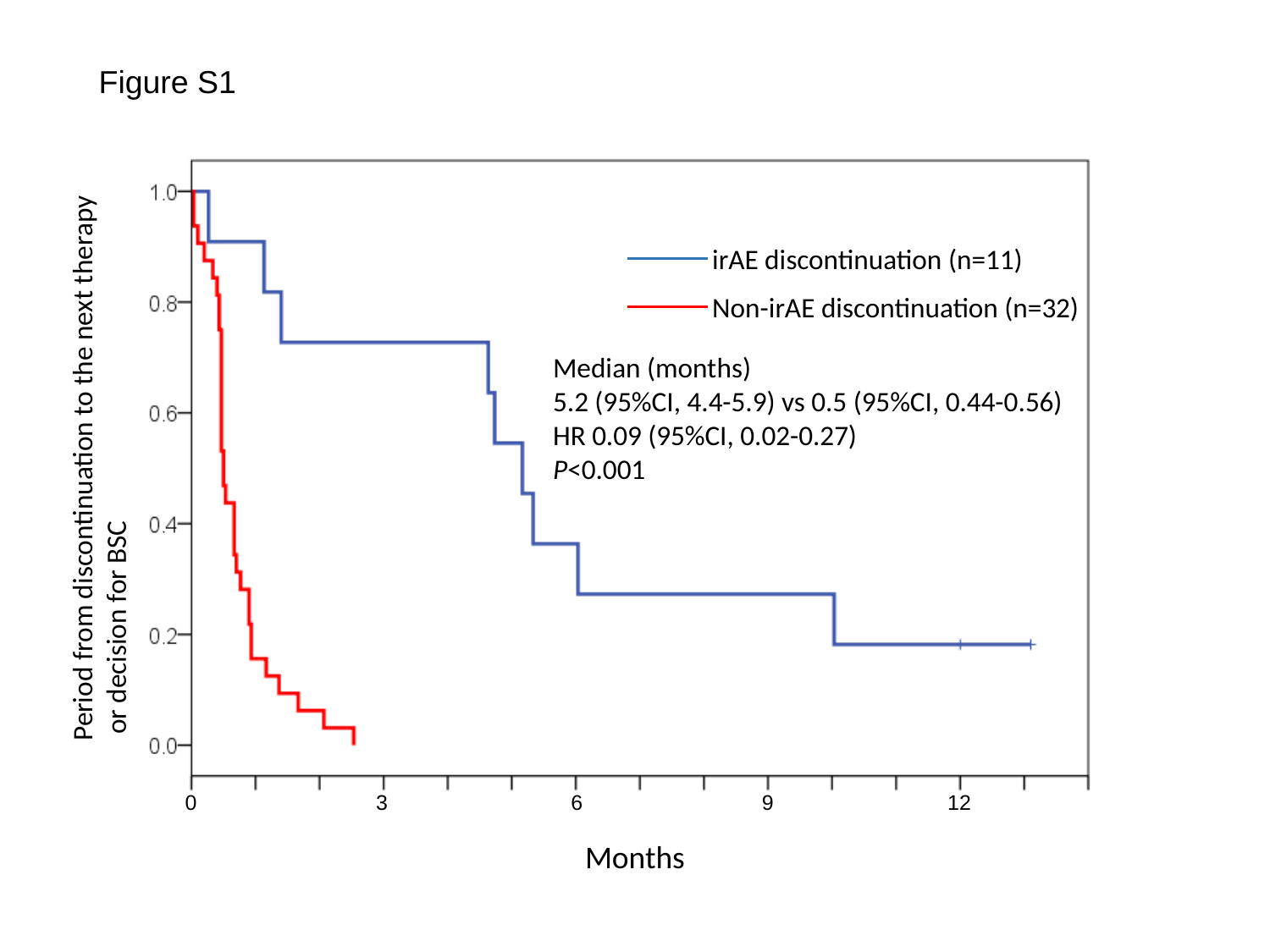

Figure S1
irAE discontinuation (n=11)
Non-irAE discontinuation (n=32)
irAE discontinuation (n=11)
Non-irAE discontinuation (n=32)
Median (months)
5.2 (95%CI, 4.4-5.9) vs 0.5 (95%CI, 0.44-0.56)
HR 0.09 (95%CI, 0.02-0.27)
P<0.001
Period from discontinuation to the next therapy
 or decision for BSC
0
3
6
9
12
Months
